# The Treatment of Small Pox; Protective and Restorative

**Published:** 1873-07

**Authors:** Melville E. Webb


### The Treatment of Small Pox; Protective and Restorative.

By Melville E. Webb, M. D., in a Report on the late Epidemic in Boston.

The following is an extract from a report read before the Massachusetts Medical Society at its meeting June 3d, and taken from the *Boston Morning Journal*:

The number of cases reported during the time embraced in my report was 3,722; the number of deaths, 1,026, or a per cent. of 27.56. These figures are sufficiently large to show the severity of the epidemic, exceeding the percentage of either New York or Pennsylvania death rates. Its usual virulence is also shown by the large number of hœmorraghic form or 7.58 per cent., all of which proved fatal. There was a still larger proportion among those not removed, but allowing the same per cent. for the whole number of cases, we have 282 deaths from this most fatal form of the disease. It occurred most frequently in vitiated constitutions, though that was not always the case. Male and female suffered equally. The middle-aged seemed more liable to this form than the old, while it has been very rare in children, in fleshy people rather than in those emaciated.

The remarkable activity of the poison was still farther attested by the unprecedented large number of recurrent cases, having personally witnessed thirty-eight cases who presented scars of former victories, three of whom had the disease twice within three months. Of those treated in the three hospitals in city limits, sixty per cent. were American born, fifteen per cent. from the British provinces, fifteen per cent. from Ireland, and ten per cent. from all other parts of the world, the death rate being about in the same proportion. The number of males treated was 538, the number of deaths 127—per cent. 23.60; number of females treated 180; number of deaths 58—per cent. 18.82, showing a difference of 4.77 per cent! Nearly one-half those unvaccinated died; of those vaccinated 29.21 per cent. died; of those revaccinated the death rate was 17.21 per cent. As showing the value of the vaccine scar, out of 413 treated in the hospitals, 53 died ; out of 103 having two scars, eight died ; out of 36 having three or four scars, none died ; of those having five scars, two died.

Vaccinnation is the only preventive known, but has not proved an entire safeguard, as proved by the number of recurrent cases among those vaccinnated in infancy. From the fact that those vaccinated and revaccinated have had the small pox, the conclusion must be arrived at that there are persons so succeptible to the influences of variola poison that no amount of vaccination, or previous attacks would prevent the disease if these persons were exposed to the contagion. As regards the scar, from many observations among patients, doctors and nurses, the conclusion is reached that vaccination may protect without any external evidence whatever. A scar is not a test of thoroughness or immunity. Tne protective power of animal over humanized virus is very much greater.

The treatment best calculated for the cure of the infection is to have a hospital located on high, dry and healthy land, with high, well-ventilated and properly warmed rooms, insuring comfort and happiness to the patients. A proper classification of the disease should be rendered practicable where the most delicate and sensitive can be so separated from the wards that they will not be continually shocked by scenes of suffering and death. Most of the patients are in the second stage, or that of erruption, on admission, and require little or no treatment except an opiate at night (as sleep is of great importance,) and some mild laxity to keep the bowels in soluble condition.

In the severer forms quinine has been given early and through every stage of the disease, thus keeping the temperature low and harboring strength for the superative stage. During the secondary fever quinine in large doses is given, milk punch, brandy, beef tea, and opiates, generally sulphate of morphine. Patients never have been without milk and have taken it at pleasure. Grapes, oranges, apples, etc., have been rationed out daily in all stages of the disease. In the hemorrhagic form the same course has been followed, with the addition of ergot, muriated tincture of iron, tanic acid and other astringents; but none of them have been of benefit.

Complications of the disease were treated upon general principles, never forgetting that the vital powers must be sustained. Ulcerations of the cornea were treated by frequent cleansing of the eyes, applying weak astringent lotions and keeping the pupils well dilated with atrophine. In the third stage it has been considered as a cry of debilitated nature, necessary to give milk-punch and beef tea. To prevent pitting, various methods have been tried. If the ulcerations extend deeply into the skin, nothing will prevent cicatrices remaining. Latterly thc treatment was emollient applications and frequent sponging with tepid water.

The hospital solution to prevent itching was carbolic acid half a drachm, glycerine half an ounce, with olive oil, mix and apply with a brush. The disinfectant used was carbolic acid, the best in destroying all odor. The wards were sprinkled with the solution; it was applied to the beds and bedding in a powder made by rubbing up the acid with magnesia. The earth and water closets were kept free of odor by lavish use of carbolic acid. Bromo-chloralum has the advantage of being nearly odorless, and in private rooms, where there was only one patient, answered every purpose; but in wards it is inferior to carbolic acid. Fumigation has been performed by burning sulphur and strongly saturating the beds and bedding with the fumes of sulphuric acid. The necessity of a good, permanent and well organized hospital, always at hand for an emergency, cannot be too strongly urged. A good hospital, maintained for five years, even at an expense of $5,000 or $10,000 a year would be economy. The past epidemic teaches the necessity of a competent Board of Health, unbiased by political tinkerers or party influence.

